# Effects of Copra Meal Inclusion Level in Growing-Finishing Pig Diets Containing β-Mannanase on Growth Performance, Apparent Total Tract Digestibility, Blood Urea Nitrogen Concentrations and Pork Quality

**DOI:** 10.3390/ani10101840

**Published:** 2020-10-09

**Authors:** Jae-Cheol Jang, Dong Hyuk Kim, Jin Su Hong, Young Dal Jang, Yoo Yong Kim

**Affiliations:** 1 Department of Agricultural Biotechnology, and Research Institute of Agriculture and Life Sciences, Seoul National University, Seoul 08826, Korea; swanjchang@gmail.com (J.-C.J.); kaiwen84@hanmail.net (D.H.K.); nutrihong@naver.com (J.S.H.); youngdal.jang@uwrf.edu (Y.D.J.); 2 Department of Animal and Food Science, University of Wisconsin-River Falls, River Falls, WI 54022, USA

**Keywords:** blood urea nitrogen, β-mannanase, copra meal, growing-finishing pigs, growth performance, digestibility, pork quality

## Abstract

**Simple Summary:**

The increasing demand and production of ethanol have led to an increase in corn price, which has resulted in increased animal feed cost. Alternative feed ingredients such as copra meal have attracted great attention in the feed and swine industry due to their comparable nutritional values and price compared to conventional swine feed ingredients, such as corn and soybean meal. The current study aims at demonstrating the effect of copra meal inclusion with β-mannanase on growth performance, nutrient digestibility, blood urea nitrogen concentrations, and pork quality of growing-finishing pigs. Our result suggested that copra meal could have the potential to be used in the swine diets up to 12% when the diets were supplemented with 800 IU of β-mannanase per kg diet.

**Abstract:**

This experiment was conducted to evaluate the effects of copra meal (CM) inclusion level on the growth performance, apparent total tract digestibility (ATTD), blood urea nitrogen (BUN) concentrations, and pork quality of growing-finishing pigs fed diets containing β-mannanase. Eighty crossbred pigs with average body weight (BW) of 27.22 ± 0.09 kg were allotted to five dietary treatments with four pigs per pen and four replicates per treatment based on sex and BW. The dietary treatments were: (1) NC: negative control, corn-soybean meal (SBM) based basal diet, (2) PC: positive control, basal diet + 0.10% β-mannanase (800 IU/ kg diet), (3) CM6: PC diet with 6% CM inclusion, (4) CM12: PC diet with 12% CM inclusion, and (5) CM18: PC diet with 18% CM inclusion in a three-phase feeding program (growing: 0–6 weeks, finishing I: 7–9 weeks, and finishing II: 10–12 weeks). The quadratic responses were observed in the BW at six weeks (*p* < 0.05), ADG in the growing phase (0–6 weeks; *p* < 0.05), and ADFI in the finishing phase with a tendency (7–12 weeks; *p* = 0.06) as the inclusion level of CM increased. However, the BW at 12 weeks (linear, *p* < 0.05 and quadratic, *p* = 0.06), the overall ADG (0–12 weeks; linear and quadratic, *p* < 0.05), and the G:F ratio in the finishing (7–12 weeks; linear, *p* < 0.05) and overall (0–12 weeks; linear, *p* < 0.05) phases decreased with increasing levels of CM in the diets. The ATTD of crude protein (linear, *p* < 0.05), crude fiber (linear, *p* < 0.05), and ash (linear, *p* < 0.05) decreased linearly as the inclusion level of CM increased. The BUN concentrations increased linearly with increasing levels of CM in the diets at 12 weeks of the experiment (*p* < 0.05). As the inclusion level of CM increased, TBARS value at d 3 post-mortem (linear, *p* = 0.07) tended to increase, whereas initial loin pH at 1 h post-mortem decreased (linear and quadratic, *p* < 0.05) with no difference in ultimate loin pH at 24 h post-mortem. These results indicated that CM inclusion up to 12% in the growing-finishing pig diets with β-mannanase did not affect growth performance, nutrient utilization, and pork quality whereas 18% CM inclusion to the diets could negatively impact nutrient digestibility, BUN concentrations, and thereby growth performance.

## 1. Introduction

Copra meal (CM) is a co-product of coconut oil production, which is a residue after oil extraction from the meat of coconut. CM has been of great interest as an alternative feed ingredient that can be utilized in the swine diets [1,2]. The CM contains 15–26% of protein, which is greater than corn and wheat bran [3,4]. However, high concentrations of non-starch polysaccharides [5] and the relatively poor digestibility of amino acids could limit the usage of CM in the swine diets [6,7]

In the CM, one of the major non-starch polysaccharides (NSP) is β-mannan, which represents 25–30% of NSP in CM [1]. The β-mannan is a polysaccharide that comprises β-1, 4-mannopyranosyl residues or mannose residues substituted by single units of α-1, 6-linked galactose [8]. Several studies reported that the β-mannan reduced the rate of glucose absorption, nitrogen retention, and fat absorption of pigs [9,10,11]. It has been reported that the inclusion of β-mannanase in the swine diets improved nutrient utilization by hydrolyzing β-mannan and galactomannan to a low molecule polysaccharide or monosaccharide, which is mannose [12]. Although a recent study reported that the inclusion of CM up to 25% had no detrimental effects on the growth performance and pork quality compared to those of pigs fed the control diet containing 5% CM when supplemented with β-mannanase [13], further demonstration is needed to determine an optimum inclusion level of CM in corn-SBM based diets for growing-finishing pigs. Therefore, the objective of this study was to evaluate the effect of increasing levels of CM in the growing-finishing pig diets containing β-mannanase on growth performance, apparent total tract digestibility (ATTD), blood urea nitrogen (BUN) concentrations, and pork quality.

## 2. Materials and Methods

### 2.1. Animal Care

This experimental protocol was approved by the Seoul National University Institutional Animal Care and Use Committee (SNU-160613-10).

### 2.2. Experimental Design, Diets and Animal Management

A total of 80 growing pigs ([Yorkshire × Landrace] × Duroc, 27.22 ± 0.09 kg of initial BW) were allotted to 5 dietary treatments with 4 pigs per pen (2 barrows and 2 gilts), and 4 replicates per treatment in a randomized complete block design, using the Experimental Animal Allotment Program developed by Kim and Lindemann, (2007). The dietary treatments were: (1) NC: negative control, corn-soybean meal (SBM) based basal diet, (2) PC: positive control, basal diet + 0.10% β-mannanase (800 IU/kg diet), (3) CM6: PC diet with 6% CM inclusion, (4) CM12: PC diet with 12% CM inclusion, and (5) CM18: PC diet with 18% CM inclusion. Copra meal was added to the diets by replacing corn and SBM with adjustments of the other feed ingredients to obtain similar nutrient content in the diets across the treatments. The analyzed chemical composition of CM was presented in the Table 1. Experimental diets were fed to growing-finishing pigs with 3 phases (growing phase = 0 to 6 weeks; finishing I phase = 7 to 9 weeks; finishing II phase = 10 to 12 weeks, respectively, Table 2, Table 3 and Table 4). All nutrients were met or exceeded requirements of NRC [4]. The metabolizable energy (ME) value [14] and calcium and phosphorus compositions [15,16] of CM were adapted from previous studies. The commercial β-mannanase (800,000 U of β-mannanase/kg; Patent, 10-0477456-0000; CTC bio^®^ Inc.) used in this study was a product of *Bacillus subtilis* (WL-7) fermentation grown on Luria broth (CTC Bio, Inc., Seoul, Republic of Korea). The ingredient (CM), diet formulations and chemical composition were presented in Table 1, Table 2, Table 3 and Table 4. 

All pigs were housed in an environmentally controlled building with a half-slotted concrete floor in growing (1.26 × 2.55 m^2^) and finishing (1.60 × 3.00 m^2^) phases. Each pen was equipped with a feeder and a nipple drinker to provide ad libitum access. Body weight and feed intake were recorded at 0, 6, and 12 weeks to calculate average daily gain (ADG), average daily feed intake (ADFI), and gain-to-feed ratio (G:F).

### 2.3. Apparent Total Tract Digestibility

For apparent total tract digestibility (ATTD) estimation, a total of fifteen barrows (54.13 ± 1.59 kg of initial BW) were allotted to 5 treatments with 3 replicates in a completely randomized design and housed in individual metabolic crates (0.93 × 1.53 m^2^). The experimental period consists of an initial 7-d adaptation period followed by a 5-day collection period. The amount of feed intake per pig was calculated as approximately 2.5 times the estimated energy requirement for maintenance in accordance with NRC, [20], and divided into 2 equal meals and fed to pigs at 07:00 and 19:00.

Feces was collected according to the marker-to-marker procedure using 2 g of ferric oxide (Fe_2_O_3_) and chromium oxide (Cr_2_O_3_) in the first and last meals as an indigestible marker, respectively. During the collection period, fecal samples were weighed, pooled, sealed in plastic bags, and frozen at −20 °C. At the end of the collection period, feces were thawed and dried in a forced-air drying oven at 60 °C for 72 h, then ground through a 1-mm screen using a Wiley mill (Thomas Model 4 Wiley Mill; Thomas Scientific, Swedesboro, NJ, USA) for chemical analysis. 

The ingredient (CM), experimental diets and feces were analyzed according to the methods of AOAC [21] for dry matter (method 930.15), crude protein (method 990.03), ether extract (method 920.39), crude fiber (method 978.10), and ash (method 942.05). The gross energy (GE) in CM was measured using bomb calorimeter (Parr 1261, Parr Instruments, IL, USA). 

### 2.4. Blood Urea Nitrogen

Blood samples were collected from jugular vein from 5 pigs per treatment selected based on body weight using a disposable vacutainer tube (Becton Dickinson, Franklin, NJ, USA) at 3, 6, and 12 weeks for blood urea nitrogen (BUN) analysis. After centrifugation (3000× *g* for 15 min at 4 °C; 5810R, Eppendorf AG, Hamburg, Germany), serum was carefully separated to plastic vials and stored at −20 °C until BUN analysis. Total BUN concentrations were analyzed using blood analyzer (Ciba-Corning model, Express Plus, Ciba Corning Diagnostics Co., MA, USA).

### 2.5. Pork Quality and Thiobarbituric Acid Reactive Substance (TBARS) Assay

After the feeding trial, 20 pigs (4 pigs per treatment; average BW: 106.73 ± 6.53 kg) were selected based on BW and sex and slaughtered with approved procedure at slaughterhouse. The longissimus muscle samples from 10th rib area were collected to evaluate pork quality. The muscle pH and color (International Commission on Illumination L^*^, a^*^, and b^*^ values representing lightness, redness, and yellowness, respectively) were measured at 1, 2, 5, 12, and 24 hours post-mortem by pH meter (Bechman Coulter Φ 500 Series, CA, USA) and Chromameter (CR-508i, Minolta Camera Co., Osaka, Japan), respectively. The loin samples were analyzed for TBARS at 1, 3, and 7 days post-mortem based on the spectrophotometric method established by Tarladgis et al. [22]. The extent of lipid oxidation was measured using the 2-thiobarbituric acid reactive substances (TBARS) method. Each sample (pork loin, 5 g) was homogenized with 15 mL deionized distilled water and 50 µL butylated hydroxyanisole 7.2% (Sigma-Aldrich, Saint Louis, MO, USA) for 15 s. Then, the 2 mL of homogenates were moved to test tubes (15 mL) and mixed with 2-thiobarbituric acid (TBA, 4 mL) and trichloroacetic acid (20 mM of TBA in 15% trichloroacetic acid). The solution was vortexed (30 s), and incubated in a boiling water bath (15 min). The sample was then cooled in cold water (10 min), and centrifuged (2500× *g* for 15 min at 4 °C). The absorbance of the supernatant was measured using a spectrophotometer (UV-240, Shimadzu Co., Kyoto, Japan) at 532 nm. The unit of TBARS values were mg malondialdehyde per kg sample.

### 2.6. Data and Statistical Analyses

A total of 3 pigs (1 pig and 2 pigs in NC and CM18 treatments, respectively) were excluded from the feeding trial due to abnormal growth and ADFI data were corrected using Feed Intake Correction Software [23] where ADFI of the pen was adjusted considering energy for the maintenance and gain of the excluded pigs. All data for growth performance (BW, ADG, ADFI, and G:F), ATTD, BUN concentrations, and pork quality were analyzed by ANOVA using MIXED procedure of SAS version 9.4 (SAS Institute Inc., Cary, NC, USA). Orthogonal polynomial contrasts were used to evaluate linear and quadratic responses with increasing levels of CM in the diets. The pen was served as the experimental unit for the performance data while the individual pig was served as the experimental unit in the ATTD, BUN concentrations, and pork quality. A single degree of freedom contrast was performed for the comparison between the NC and PC treatments to verify the β-mannanase effect. The LSMEANS procedure of SAS was used to estimate and report mean values. The significance level was set at *p* < 0.05, and trends are reported when 0.05 ≤ *p* ≤ 0.10.

## 3. Results and Discussion

### 3.1. Growth Performance 

The analyzed chemical composition of CM used in this experiment was in accordance with previous studies ([14,24], Table 1). The PC treatment tended to have a greater G:F than the NC treatment (*p* = 0.10) in the growing phase (0–6 weeks, Table 5). Similar numerical differences were observed on the G:F in the finishing phase. This result agrees with Jo et al. [25] who reported an improvement in growth performance of growing-finishing pigs fed corn-SBM-based diets supplemented with 400 IU β-mannanase/kg diet. Regarding the effect of inclusion level of CM, quadratic responses were observed in the BW at 6 weeks (*p* < 0.05), ADG in the growing phase (0–6 weeks; *p* < 0.05), and ADFI in the finishing phase (7–12 weeks; *p* = 0.06) as the inclusion level of CM increased with the greatest values between CM6 and CM12 treatments. However, linear and quadratic reductions were observed in the BW at 12 weeks (linear, *p* < 0.05 and quadratic, *p* = 0.06) and the overall ADG (0–12 weeks; linear and quadratic, *p* < 0.05) with increasing level of CM in the diets. The G:F in the finishing and overall phases decreased linearly with increasing level of CM in the diets (*p* < 0.05). These results indicated that over 12% CM inclusion could result in growth depression even though 800 IU of β-mannanase per kg diet was supplemented to the diets. This result is in a good agreement with previous studies reporting that high level of CM inclusion in the diets could reduce growth performance of pigs [2,26]. O’Doherty and McKeon [26] suggested that high fiber content in the CM could decrease the passage rate of digesta in the gastrointestinal tract, leading to decreased nutrient digestibility, in turn resulting in reduced growth rate. In contrast, Kim et al. [13] reported that up to 25% CM inclusion to growing-finishing pig diets supplemented with 800 IU of β-mannanase per kg diet had no detrimental effect on growth performance, which may indicate that 800 IU of β-mannanase per kg diet could be sufficient to degrade the β-mannans present in the diet containing CM up to 20%. Based on the results of Kim et al. [13], 800 IU of β-mannanase per kg diet was added to all experimental diets in the current study. However, the current study had the lowest growth performance in the CM18 treatments. This discrepancy may be attributed to the differences in the ingredient composition of the diets between the current study and Kim et al. [13]. 

### 3.2. Apparent Total Tract Aigestibility

In the current study, the ATTD of crude protein (linear, *p* < 0.05), crude fiber (linear, *p* < 0.05), and ash (linear, *p* < 0.05) decreased linearly as the inclusion level of CM increased in the diet with the greatest reductions between CM12 and CM18 treatments (Table 6). The CM contains about 2.8 times total dietary fiber (TDF) compared with SBM (46.9% in CM and 16.9% in SBM; NRC, [4]). The high content of TDF in the diets is associated with blocking the access of digestive enzymes to the cell contents [27] resulting in reduced nutrient digestibility [6,26,28]. O’Doherty and McKeon [26] highlighted that increasing levels of CM in the diets linearly reduced crude protein digestibility due to the negative correlation between amino acid digestibility and neutral detergent fiber content in the diets [29,30]. The CM also contains high β-mannan content [17] and has high water holding capacity [31], which cause swelling in the gastrointestinal tract of pigs, resulting in reduced fiber digestibility [32]. Therefore, increasing levels of CM in the diet had a linear adverse impact on crude protein, crude fiber, and ash digestibility. This may attributed to the overwhelming level of β-mannans in the diets such that the activity of β-mannanase supplemented in the diet may not be sufficient to degrade all β-mannans present in the diets. This impact was more obvious in 18% CM treatment.

### 3.3. Blood Urea Nitrogen Concentrations

There were no significant differences in the BUN concentrations except for a linear increase at 12 weeks of the experiment (*p* < 0.05; Table 7) with the greatest increase between CM12 and CM18 treatments. Similar numerical trends were observed at six weeks of the experiment. This result agrees with a previous study by Kim et al. [13] who reported that increasing level of CM in the diets with β-mannanase decreased the BUN concentrations of pigs. The BUN is inversely correlated to protein quality and utilization [33], implying that an increase in BUN concentrations indicate decreased efficiency of nitrogen utilization and lean gain [34]. Therefore, increasing levels of CM linearly decreased protein efficiency. Especially, 18% CM inclusion may reduce protein utilization efficiency, although β-mannanase was supplemented in the diets. In addition, this may explain the decreased growth performance and crude protein digestibility in the current study.

### 3.4. Pork Quality (TBARS Value, pH, and Color of Loin)

The TBARS values at d 3 post-mortem tended to increase linearly (*p* = 0.07) as the inclusion level of CM increased (Table 8). Babatunde et al. [35] reported the fat composition of diets could affect composition of accumulated fat in the body of pigs. Several studies indicated that inclusion of CM in the diet increased saturated fatty acid (myristic acid; C14:0) level in the backfat of finishing pigs [2,36]. However, in the current study, the TBARS values increased with increasing level of CM inclusion in the diets. The CM contains 70–80% of total saturated fatty acids (lauric and myristic acids; NRC, [4]) whereas soy oil contains 80–85% of total unsaturated fatty acids [37]. We speculate that increasing levels of soy oil in the experimental diets associated with increasing levels of CM inclusion due to low metabolizable energy value of the CM may alter the fatty acid composition in pork, leading to the accumulation of more unsaturated fatty acids, which are susceptible to lipid peroxidation during storage.

There was a decrease in the loin pH at 1 h post-mortem (*p* < 0.05, linear and quadratic) whereas ultimate pH at 24 h post-mortem and loin color were not different among the CM treatments (Table 9). The change of loin pH is an important factor to determine the quality of pork because it is correlated with freshness, water holding capacity, tenderness, binding ability, pork color, texture, and shelf life [38,39]. This results may indicate that CM inclusion up to 18% with β-mannanase supplementation had no adverse effect on pork quality (loin pH and color), which is in an agreement with Kim et al. [13], who reported that there were no differences in loin pH and color when the pigs were fed diets containing up to 25% CM with β-mannanase supplementation.

## 4. Conclusions

The CM inclusion up to 12% in the growing-finishing pig diets with β-mannanase supplementation did not negatively affect growth performance, protein and fiber digestibility, or BUN concentrations of pigs. However, CM inclusion at 18% could negatively impact growth rate, protein, and fiber utilization even though β-mannanase was supplemented in the diets. The CM inclusion may have no detrimental effects on pork quality. Therefore, CM could have the potential to be used in the swine diet up to 12% when the diets were supplemented with 800 IU of β-mannanase per kg diet.

## Figures and Tables

**Table 1:** The chemical composition of copra meal (%, as-fed basis).

Item	Copra Meal
Dry matter	90.3
Gross Energy, kcal/kg	4088
Crude protein	22.2
Either extract	3.84
Crude fiber	11.1
Ash	5.8

**Table 2:** The formula and chemical composition of experimental diets in growing phase.

Ingredients	Treatment ^1^				
	NC	PC (CM0)	CM6	CM12	CM18
Corn, yellow dent	69.40	69.18	64.29	59.42	54.44
SBM (48% CP)	28.42	28.46	26.50	24.55	22.62
Copra meal	0.00	0.00	6.00	12.00	18.00
Tallow	0.10	0.18	0.88	1.57	2.25
L-Lysine HCl	0.02	0.02	0.17	0.30	0.42
Tricalcium phosphate	1.00	1.00	1.00	1.00	1.00
Limestone	0.46	0.46	0.46	0.46	0.46
Vitamin premix ^2^	0.10	0.10	0.10	0.10	0.10
Trace mineral premix ^3^	0.10	0.10	0.10	0.10	0.10
Salt	0.30	0.30	0.30	0.30	0.30
Polymyxin ^4^	0.10	0.10	0.10	0.10	0.10
β-mannanase ^5^	0.00	0.10	0.10	0.10	0.10
Total	100.00	100.00	100.00	100.00	100.00
Chemical composition ^6^					
ME ^7^, kcal/kg	3300.54	3300.67	3300.51	3300.56	3300.47
Crude protein, %	18.40	18.40	18.40	18.40	18.40
SID ^8^ lysine, %	1.00	1.00	1.00	1.00	1.00
SID ^8^ methionine, %	0.30	0.30	0.30	0.30	0.30
Total Ca, %	0.66	0.66	0.66	0.66	0.66
STTD ^8^ P, %	0.30	0.30	0.30	0.31	0.32
Total NSP ^9^, %	11.49	11.48	13.50	15.51	17.52
β-mannan ^9^, %	0.43	0.44	1.91	3.38	4.85

^1^ NC = negative control (corn-soybean meal-based diet); PC = positive control (basal diet + 0.10% β-mannanase, 800 IU/kg diet); CM6 = PC diet + 6% CM; CM12 = PC diet + 12% CM; CM18 = PC diet + 18% CM. ^2^ Provided per kg of diet: vitamin A, 16,000 IU; vitamin D_3_, 3200 IU; vitamin E, 35 IU; vitamin K_3_, 5 mg; riboflavin, 6 mg; pantothenic acid, 16 mg; niacin, 32 mg; d-biotin, 128 μg, vitamin B_12_, 20 μg. ^3^ Provided per kg of diet: Fe, 281 mg; Cu, 288 mg; Mn, 49 mg; I, 0.3 mg; Se, 0.3 mg. ^4^ Colistin: 110 mg/kg. ^5^ CTCzyme^®^: CTC bio Inc. Seoul, Republic of Korea. ^6^ Calculated values. ^7^ Metabolizable energy. ^8^ SID = Standardized ileal digestible; STTD = Standardized total tract digestible. ^9^ NSP = non-starch polysaccharides. Total NSP and β-mannan contents in the diet were calculated based on Dierick [17], Knudsen [5], Hsiao et al. [18], and Jaworski and Stein [19].

**Table 3:** The formula and chemical composition of experimental diets in finishing I phase.

Ingredients	Treatment ^1^				
	NC	PC (CM0)	CM6	CM12	CM18
Corn, yellow dent	74.11	73.91	69.32	64.68	60.05
SBM (48% CP)	21.09	21.12	19.13	17.14	15.16
Wheat bran	3.00	3.00	3.00	3.00	3.00
Copra meal	0.00	0.00	6.00	12.00	18.00
Soy oil	0.08	0.15	0.71	1.29	1.86
L-Lysine HCl	0.00	0.00	0.04	0.09	0.13
Tricalcium phosphate	0.80	0.80	0.74	0.70	0.66
Limestone	0.42	0.42	0.46	0.50	0.54
Vitamin premix ^2^	0.10	0.10	0.10	0.10	0.10
Trace mineral premix ^3^	0.10	0.10	0.10	0.10	0.10
Salt	0.30	0.30	0.30	0.30	0.30
β-mannanase ^4^	0.00	0.10	0.10	0.10	0.10
Total	100.00	100.00	100.00	100.00	100.00
Chemical composition ^5^					
ME ^6^, kcal/kg	3300.21	3300.16	3300.23	3300.25	3300.10
Crude protein, %	14.50	14.50	14.50	14.50	14.50
SID ^7^ lysine, %	0.75	0.75	0.75	0.75	0.75
SID ^7^ methionine, %	0.23	0.23	0.23	0.23	0.23
Total Ca, %	0.52	0.52	0.52	0.52	0.52
STTD ^7^ P, %	0.24	0.24	0.24	0.25	0.26
Total NSP ^8^, %	11.62	11.61	13.64	15.67	17.70
β-mannan ^8^, %	0.35	0.36	1.82	3.29	4.76

^1^ NC = negative control (corn-soybean meal-based diet); PC = positive control (basal diet + 0.10% β-mannanase, 800 IU/kg diet); CM6 = PC diet + 6% CM; CM12 = PC diet + 12% CM; CM18 = PC diet + 18% CM. ^2^ Provided per kg of diet: vitamin A, 16,000 IU; vitamin D_3_, 3200 IU; vitamin E, 35 IU; vitamin K_3_, 5 mg; riboflavin, 6 mg; pantothenic acid, 16 mg; niacin, 32 mg; d-biotin, 128 μg, vitamin B_12_, 20 μg. ^3^ Provided per kg of diet: Fe, 281 mg; Cu, 288 mg; Mn, 49 mg; I, 0.3 mg; Se, 0.3 mg. ^4^ CTCzyme^®^: CTC bio Inc. Seoul, Republic of Korea. ^5^ Calculated values. ^6^ Metabolizable energy. ^7^ SID = Standardized ileal digestible; STTD = Standardized total tract digestible. ^8^ NSP = non-starch polysaccharides. Total NSP and β-mannan contents in the diet were calculated based on Dierick [17], Knudsen [5], Hsiao et al. [18], and Jaworski and Stein [19].

**Table 4:** The formula and chemical composition of experimental diets in finishing II phase.

Ingredients	Treatment ^1^				
	NC	PC (CM0)	CM6	CM12	CM18
Corn, yellow dent	78.75	78.57	73.91	69.26	64.69
SBM (48% CP)	14.51	14.55	12.58	10.60	8.59
Wheat bran	5.04	5.04	5.04	5.04	5.04
Copra meal	0.00	0.00	6.00	12.00	18.00
Soy oil	0.10	0.16	0.74	1.32	1.88
L-Lysine HCl	0.00	0.00	0.04	0.08	0.13
Tricalcium phosphate	0.58	0.55	0.51	0.50	0.44
Limestone	0.52	0.53	0.58	0.60	0.63
Vitamin premi ^2^	0.10	0.10	0.10	0.10	0.10
Trace mineral premi ^3^	0.10	0.10	0.10	0.10	0.10
Salt	0.30	0.30	0.30	0.30	0.30
β-mannanas ^4^	0.00	0.10	0.10	0.10	0.10
Total	100.00	100.00	100.00	100.00	100.00
Chemical composition ^5^					
ME ^6^, kcal/kg	3300.00	3300.13	3300.11	3300.12	3300.22
Crude protein, %	12.80	12.80	12.80	12.80	12.80
SID ^7^ lysine, %	0.61	0.61	0.61	0.61	0.61
SID ^7^ methionine, %	0.20	0.20	0.20	0.20	0.20
Total Ca, %	0.46	0.46	0.46	0.46	0.46
STTD ^7^ P, %	0.21	0.21	0.21	0.22	0.23
Total NSP ^8^, %	11.52	11.51	13.54	15.57	17.60
β-mannan ^8^, %	0.28	0.28	1.75	3.22	4.69

^1^ NC = negative control (corn-soybean meal-based diet); PC = positive control (basal diet + 0.10% β-mannanase, 800 IU/kg diet); CM6 = PC diet + 6% CM; CM12 = PC diet + 12% CM; CM18 = PC diet + 18% CM. ^2^ Provided per kg of diet: vitamin A, 16,000 IU; vitamin D_3_, 3200 IU; vitamin E, 35 IU; vitamin K_3_, 5 mg; riboflavin, 6 mg; pantothenic acid, 16 mg; niacin, 32 mg; d-biotin, 128 μg, vitamin B_12_, 20 μg. ^3^ Provided per kg of diet: Fe, 281 mg; Cu, 288 mg; Mn, 49 mg; I, 0.3 mg; Se, 0.3 mg. ^4^ CTCzyme^®^: CTC bio Inc. Seoul, Republic of Korea. ^5^ Calculated values. ^6^ Metabolizable energy. ^7^ SID = Standardized ileal digestible; STTD = Standardized total tract digestible. ^8^ NSP = non-starch polysaccharides. Total NSP and β-mannan contents in the diet were calculated based on Dierick [17], Knudsen [5], Hsiao et al. [18], and Jaworski and Stein [19].

**Table 5:** Effects of copra meal (CM) inclusion level in the diet with β-mannanase on growth performance of growing-finishing pigs ^1^.

Items	Treatment ^2^					SEM ^3^	*p*-Values ^4^		
	NC	PC (CM0)	CM6	CM12	CM18		NC vs. PC	Lin	Quad
**Body weight** **,** **k** **g**									
Initial	26.80	27.02	27.02	27.00	26.98	0.131	0.89	0.29	0.69
6 weeks	64.30	64.44	67.07	66.95	62.90	3.088	0.54	0.39	0.02
12 weeks	101.52	104.12	106.23	104.31	99.22	2.400	0.21	0.05	0.06
**ADG** **, g/d**									
0–6 weeks	893	891	954	951	856	37.7	0.66	0.40	0.02
7–12 weeks	866	923	911	869	845	35.4	0.62	0.13	0.87
0–12 weeks	879	907	932	910	850	19.1	0.23	0.04	0.04
**ADFI** **, g/d**									
0–6 weeks	1933	1861	1949	1957	2015	113.1	0.65	0.28	0.87
7–12 weeks	2868	2795	3020	3070	2886	99.8	0.21	0.47	0.06
0–12 weeks	2400	2328	2485	2513	2451	90.5	0.61	0.33	0.24
**G:F**									
0–6 weeks	0.462	0.481	0.492	0.493	0.430	0.025	0.10	0.17	0.14
7–12 weeks	0.303	0.333	0.302	0.284	0.292	0.014	0.61	0.04	0.15
0–12 weeks	0.367	0.392	0.375	0.363	0.347	0.010	0.27	0.01	0.99

^1^ Growing phase = 0–6 weeks; finishing I phase = 7–12 weeks; overall phase = 0–12 weeks. ^2^ NC = negative control (corn-soybean meal-based diet); PC = positive control (basal diet + 0.10% β-mannanase, 800 IU/kg diet); CM6 = PC diet + 6% CM; CM12 = PC diet + 12% CM; CM18 = PC diet + 18% CM. ^3^ Standard error of the means. ^4^ NC vs. PC = negative control vs. positive control; Linear (Lin) and quadratic (Quad) = 0, 6, 12 and 18% CM with 0.10% (800 IU/kg diet) β-mannanase.

**Table 6:** Effects of copra meal (CM) inclusion level in the diet with β-mannanase on apparent total tract digestibility of growing pigs ^1^.

Items	Treatment ^2^					SEM ^3^	*p*-Values ^4^		
	NC	PC (CM0)	CM6	CM12	CM18		NC vs. PC	Lin	Quad
**Apparent total tract digestibility, %**									
Dry matter	88.95	88.38	89.67	89.52	89.53	0.140	0.89	0.78	0.65
Crude protein	89.82	93.82	88.92	90.06	79.73	3.113	0.78	0.01	0.28
Ether extract	68.00	77.59	79.97	81.65	64.07	9.499	0.41	0.24	0.18
Crude fiber	68.32	80.11	68.35	75.39	50.18	7.984	0.66	0.04	0.36
Ash	76.76	83.26	76.58	78.10	55.76	7.495	0.35	0.05	0.34

^1^ N = 3 individual pigs per treatment (54.13 ± 1.59 kg of initial body weight). ^2^ NC = negative control (corn-soybean meal-based diet); PC = positive control (basal diet + 0.10% β-mannanase, 800 IU/kg diet); CM6 = PC diet + 6% CM; CM12 = PC diet + 12% CM; CM18 = PC diet + 18% CM. ^3^ Standard error of the means. ^4^ NC vs. PC = negative control vs. positive control; Linear (Lin) and quadratic (Quad) = 0, 6, 12 and 18% CM with 0.10% (800 IU/kg diet) β-mannanase.

**Table 7:** Effects of copra meal (CM) inclusion level in the diet with β-mannanase on blood urea nitrogen concentrations ^1^.

Items	Treatment ^2^					SEM ^3^	*p*-Values ^4^		
	NC	PC (CM0)	CM6	CM12	CM18		NC vs. PC	Lin	Quad
**Blood urea nitrogen** **, mg/dL**									
3 week	12.14	13.31	12.20	11.87	12.09	0.671	0.82	0.26	0.39
6 week	11.36	10.05	10.76	10.54	12.12	0.825	0.21	0.13	0.60
12 week	11.05	11.35	11.51	11.53	13.80	0.687	0.57	0.03	0.13

^1^*N* = 5 individual pigs per treatment. ^2^ NC = negative control (corn-soybean meal-based diet); PC = positive control (basal diet + 0.10% β-mannanase, 800 IU/kg diet); CM6 = PC diet + 6% CM; CM12 = PC diet + 12% CM; CM18 = PC diet + 18% CM. ^3^ Standard error of the means. ^4^ NC vs. PC = negative control vs. positive control; Linear (Lin) and quadratic (Quad) = 0, 6, 12 and 18% CM with 0.10% (800 IU/kg diet) β-mannanase.

**Table 8:** Effects of copra meal (CM) inclusion level in the diet with β-mannanase on concentrations of thiobarbituric acid reactive substances (TBARS) in pork loin ^1^.

Items	Treatment ^2^					SEM ^3^	*p*-Values ^4^		
	NC	PC (CM0)	CM6	CM12	CM18		NC vs. PC	Lin	Quad
**TBARS** **, mg malondialdehyde/kg**									
1 d post-mortem	0.115	0.110	0.115	0.109	0.112	0.0022	0.21	0.92	0.72
3 d post-mortem	0.119	0.114	0.115	0.118	0.121	0.0027	0.44	0.07	0.75
7 d post-mortem	0.153	0.190	0.200	0.188	0.177	0.0146	0.60	0.48	0.51

^1^ N = 4 individual longissimus muscle (10th rib) samples per treatment. ^2^ NC = negative control (corn-soybean meal-based diet); PC = positive control (basal diet + 0.10% β-mannanase, 800 IU/kg diet); CM6 = PC diet + 6% CM; CM12 = PC diet + 12% CM; CM18 = PC diet + 18% CM. ^3^ Standard error of the means. ^4^ NC vs. PC = negative control vs. positive control; Linear (Lin) and quadratic (Quad) = 0, 6, 12 and 18% CM with 0.10% (800 IU/kg diet) β-mannanase.

**Table 9:** Effects of copra meal (CM) inclusion level in the diet with β-mannanase on pork loin pH and color 1.

Items	Treatment ^2^					SEM ^3^	*p*-Values ^4^		
	NC	PC (CM0)	CM6	CM12	CM18		NC vs. PC	Lin	Quad
**pH**									
1 h post-mortem	5.73	6.09	5.92	5.76	5.79	0.090	0.22	0.04	0.01
2 h post-mortem	5.98	6.11	5.78	5.94	5.89	0.110	0.72	0.24	0.16
5 h post-mortem	5.87	5.90	5.91	5.78	5.81	0.071	0.73	0.19	0.84
12 h post-mortem	5.72	5.78	5.73	5.70	5.81	0.066	0.27	0.80	0.17
24 h post-mortem	5.69	5.69	5.69	5.72	5.76	0.052	0.60	0.32	0.72
**Hunter L ^5^**									
1 h post-mortem	48.46	49.64	49.49	49.52	48.51	0.869	0.41	0.31	0.55
2 h post-mortem	48.00	49.54	48.64	49.45	49.21	0.941	0.85	0.95	0.56
5 h post-mortem	47.62	48.16	49.47	48.49	48.06	1.198	0.79	0.73	0.32
12 h post-mortem	46.07	48.57	49.22	47.29	47.76	1.279	0.80	0.45	0.94
24 h post-mortem	45.93	48.87	50.00	48.21	47.83	1.455	0.86	0.50	0.64
**Hunter a ^5^**									
1 h post-mortem	2.26	2.35	2.36	2.29	2.50	0.549	0.79	0.88	0.86
2 h post-mortem	3.23	2.88	2.93	2.45	2.69	0.510	0.73	0.66	0.86
5 h post-mortem	3.00	3.33	3.33	3.00	2.67	0.516	0.61	0.30	0.73
12 h post-mortem	4.29	3.66	3.71	3.67	3.15	0.571	0.53	0.57	0.65
24 h post-mortem	4.33	4.00	4.33	3.67	4.00	0.577	0.69	0.82	0.99
**Hunter b ^5^**									
1 h post-mortem	6.35	6.01	5.97	5.69	5.74	0.342	0.90	0.50	0.89
2 h post-mortem	6.66	6.18	5.92	5.92	5.83	0.382	0.86	0.54	0.82
5 h post-mortem	6.83	6.36	6.26	6.22	6.26	0.386	0.95	0.85	0.87
12 h post-mortem	7.06	6.67	6.58	6.58	6.43	0.329	0.75	0.65	0.94
24 h post-mortem	7.34	6.88	6.80	6.64	6.72	0.272	0.83	0.63	0.79

^1^ N = 4 individual longissimus muscle (10th rib) samples per treatment. ^2^ NC = negative control (corn-soybean meal-based diet); PC = positive control (basal diet + 0.10% β-mannanase, 800 IU/kg diet); CM6 = PC diet + 6% CM; CM12 = PC diet + 12% CM; CM18 = PC diet + 18% CM. ^3^ Standard error of the means. ^4^ NC vs. PC = negative control vs. positive control; Linear (Lin) and quadratic (Quad) = 0, 6, 12 and 18% CM with 0.10% (800 IU/kg diet) β-mannanase. ^5^ Hunter L = luminance or brightness (vary from black to white); a = red · green component (+a = red, −a = green); b = yellow · blue component (+b = yellow, −b = blue).
